# Understanding the impact loading characteristics of a badminton lunge among badminton players

**DOI:** 10.1371/journal.pone.0205800

**Published:** 2018-10-12

**Authors:** Wing-Kai Lam, Ki-Kwang Lee, Sang-Kyoon Park, Jaejin Ryue, Suk-Hoon Yoon, Jiseon Ryu

**Affiliations:** 1 Department of Kinesiology, Shenyang Sports Institute, Shenyang, China; 2 Li Ning Sports Science Research Center, Li Ning (China) Sports Goods Co. Ltd, Beijing, China; 3 Biomechanics & Sport Engineering Laboratory, Kookmin University, Seoul, Korea; 4 Motion Innovation Centre, Korea National Sport University, Seoul, Korea; California State University, UNITED STATES

## Abstract

**Background:**

The rapid and repetitive badminton lunges would produce strenuous impact loading on the lower extremities of players and these loading are thought to be the contributing factors of chronic knee injuries. This study examined the impact loading characteristics in various groups of badminton athletes performing extreme lunges.

**Methods:**

Fifty-two participants classified into male skilled, female skilled, male unskilled, and female unskilled groups performed badminton lunge with their maximum-effort. Shoe-ground kinematics, ground reaction forces, and knee moments were measured by using synchronised force platform and motion analysis system. A 2 (gender) x 2 (skill-level) factorial ANOVA was performed to determine the effects of different gender and different playing levels, as well as the interaction of two factors on all variables.

**Results:**

Male athletes had faster approaching speed (male 3.87 and female 1.08 m/s), longer maximum lunge distance (male 1.47 and female 1.16 m), larger maximum (male 215.7 and female 121.65 BW/s) and mean loading rate (male 178.43 and female 81.77 BW/s) and larger peak knee flexion moment (male 0.75 and female 0.69) compared with female athletes (*P* < 0.001). Unskilled athletes exhibited smaller footstrike angle (skilled 45.78 and unskilled 32.35°), longer contact time (skilled 0.69 and unskilled 0.75 s), larger peak horizontal GRF (skilled 1.61 and unskilled 2.40 BW), smaller mean loading rate (skilled 150.15 and unskilled 110.05 BW/s) and larger peak knee flexion moment (*P* < .05; skilled 0.69 and unskilled 0.75 Nm/BW) than the skilled athletes. In addition, the interaction indicated greater peak GRF impact in female unskilled athletes compared with female skilled athletes (*P* < 0.001; female skilled 2.01 and female unskilled 2.95 BW), while there was no difference between male participants (*P* > 0.05; male skilled 2.19 and male unskilled 2.49 BW).

**Conclusions:**

These data suggested that male athletes and/or unskilled athletes experience greater impact loading rates and peak knee flexion moment during lunge compared with female and skilled athletes, respectively. This may expose them to higher risk of overuse injuries. Furthermore, female unskilled athletes seemed to be more vulnerable to lower extremity injuries.

## Introduction

Badminton is a popular sport with 200 million participants worldwide [1] and it can be practiced by anyone regardless of gender and skill level. The gender and skill-level effects have been predominantly found in technical skills, anthropometry, physical performance capacities and visual searching skills, but there is a paucity of biomechanical studies concerning the differences across genders and skill levels. Compared to female badminton athletes, male athletes exhibited greater match duration, work density and rest time [2, 3] as well as better aerobic and anaerobic fitness [4–6]. Compared to less skilled badminton athletes, more skilled athletes generated quicker and more accurate response [7], faster shuttle smash velocities [8], and better aerobic and anaerobic fitness [9]. Considering that sports participation is associated with injuries, a proper understanding of biomechanics and impact characteristics among various groups of players would provide coaches and physicians insights to formulate training regime strategies to lower the risks of overuse injuries.

Badminton is an intense racket sport, with rapid jumps, lunges and turning [1,3]. The rapid and repetitive badminton lunges and jumps have been suggested to produce strenuous impact loading on the lower extremities of players, resulting in overuse knee injuries [10]. High vertical and horizontal impact forces at early contact phase would generate a high joint torque on the ligaments of the lower limbs in badminton jump and lunge [11–13], and these forces are thought to be the contributing factors of patellar tendionosis and anterior cruciate ligament (ACL) injuries [10, 14–17]. Since lunge step is one of the most frequently executed footwork drills in badminton and accounts for about 15% of the total number of movements during a single game [18, 19] and this is a particularly essential footwork that allows players to quickly move into the best position for various offensive and defensive shots, the greater attention on impact loading characteristics during lunge seems warranted.

Literature on gender studies suggested that female athletes have higher injury risks than the male counterparts, as indicated in cutting and landing manoeuvres [20–22]. However, the biomechanical characteristics of the lunge step between male and female participants have not been investigated. The findings from other non-lunging movements (cutting and landing) may not directly apply to badminton players simply due to the distinct movement characteristics and unique footstrike angles. The footstrike angle is defined as initial contact of heel strike (i.e. shoe-ground angle in sagittal plane) during lunge and a larger footstrike angle is related to higher loading rate [23]. In badminton, players often performed extreme lunges with an initial footstrike angle of more than 40 degrees [24, 25], which is much larger than in running, where the angles are approximately to 20 degrees [26]. Previous badminton studies showed differences between male and female badminton players in muscle activity pattern and bony alignment [27]. Studying the differences of male and female players would provide additional insights to understand the underlying mechanism of loading transfer to the body.

Furthermore, players of various playing levels would perform lunging with unique techniques and patterns, which might have caused to different biomechanical outcomes. Previous studies on badminton lunge found that skilled and unskilled athletes displayed distinct lunge plantar pressure profiles, showing that unskilled athletes experienced higher plantar loading in lateral forefoot but lower loading in medial forefoot compared with the skilled athletes [19]. However, impact injuries usually occur at the early (or impact) phase and the plantar pressure measuring system used in the previous study [19] fails to examine any information in the impact phase. It is suggested that ground reaction force (GRF) and knee loading can provide additional valuable information towards understanding both the mechanism and the skill level bias associated with overuse injuries.

It is recognised that extreme knee loading may be exacerbated through abnormal neuromuscular control in the transverse and frontal plane hip positions due to gender and skill level differences. Hence, the objective of this study was to examine the effect of gender and skill level on peak vertical GRF, loading rate, peak horizontal GRF, knee moment and kinematics during early stance phase of a lunge. It is hypothesized that less-skill and/or female athletes would experience a higher GRF or knee loading during an extreme badminton lunge.

## Materials and methods

### Participants

Fifty-two right-handed badminton players participated in this study. They were classified into male skilled (n = 11), female skilled (n = 11), male unskilled (n = 15), and female unskilled (n = 15) according to their gender and level of playing experience (Participants’ characteristics, see Table 1). All of the participants in the skilled groups had participated in international competitions and more than half of them achieved Korean collegiate championship titles during the year of data collection. None of the participants in the unskilled groups had taken part in any formal competition, an average of two- to three-year badminton experience and less than one-hour of playing per week. All participants were free from any lower extremity injuries for at least six months prior to the start of the study. The individual in this manuscript has given written informed consent and the testing procedure was approved by the Kookmin University Ethics Committee.

**Table 1:** Participant characteristics (raw data found in S1 File).

	Male		Female	
	Skilled	Unskilled	Skilled	Unskilled
Age (year)	20.55(0.68)	21.40(1.55)	21.91(2.55)	21.60(1.50)
Height (m)	1.78(0.06)	1.76(0.06)	1.67(0.07)*^#^	1.64(0.04) *^#^
Body mass (kg)	70.91(5.92)	66.93(5.65)	60.82(5.74)*	57.93(5.98)*^#^
Playing experience (year)	8.36(1.43)	3.20(1.01)*	10.09(1.45)*^#^	2.13(0.64)*^

*,#,^ indicate significant difference (*P* < .05) from male skilled, male unskilled and female skilled athletes, respectively.

### Apparatus and tasks

**Fig 1:**
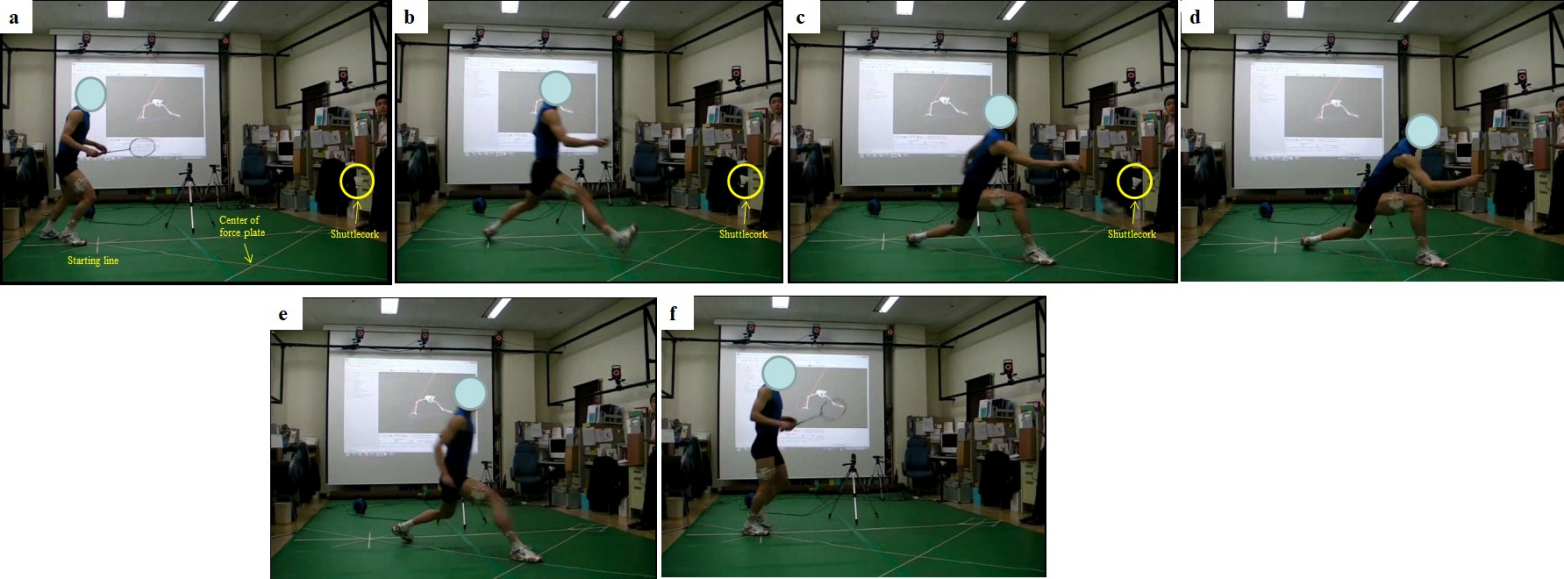
Movement sequence. a) Ready at start line, b) prepare for lunge landing, c) racket hits the shuttle cork, d) complete follow through, e) recovery to the start line and f) finish.

All participants performed five extreme lunge trials which was considered as one of the most critical manoeuvres with the greatest impact intensity among other lunge direction (Fig 1, [24]). Professional badminton mat (Li Ning CP55 Premium Court Mat, Beijing, China) was glued to the top of the force plate (Advanced Mechanical Technology Inc, Watertown, USA) and its surrounding surface to simulate the realistic shoe-ground interfaces of a standard badminton court. The synchronised force plate and an eight-camera infrared motion analysis system (Oxford Metrics Ltd, Oxford, UK) were used to collect the GRF and kinematic information of the players during each lunge trial. Both static and dynamic calibrations were performed to determine the position and orientation of the capturing volume in order to minimize the lens distortion of each camera. The cameras were aligned in a circular fashion to allow capturing of all reflective markers during lunge landing. A shuttlecock was suspended at a height of 0.6 m with a string, which was 0.6 m from the center of the force plate along the lunging direction [13].

### Procedure

Reflective markers were placed over participants’ dominant leg according to a previous segment model [13, 28]. After a standardized warm-up protocol including lower limb stretching exercises, participants familiarised themselves with the forward lunge step and tightened the lacings of the standard badminton shoes according to their individual preference for a badminton game.

Prior to actual data collection, three lunge trials with maximum-effort were used to determine the individual maximum lunge distance and respective starting position relative to the force plate. Participants holding their badminton racket were instructed to initiate the lunge movement from the start line and to extend their dominant knee as far as possible and land on the force platform whilst hitting the target shuttlecock with the standard racket (Li Ning Ultra Carbon 9000, Beijing, China) in order to simulate the actual front court shuttle-drop situation in a badminton game. After hitting the shuttlecock, participants were required to return to the start position as fast as possible (Fig 1). Successful trial consisted of maximum-effort, correct foot placement at the lunge start line, contact of the dominant leg with the center of the force plate, hit the shuttlecock and recovery to the starting point within three seconds [18, 29]. The trial was discarded if an obvious slippage and discontinuity of movement was present. Five successful lunge trials were obtained.

### Data analysis

**Fig 2:**
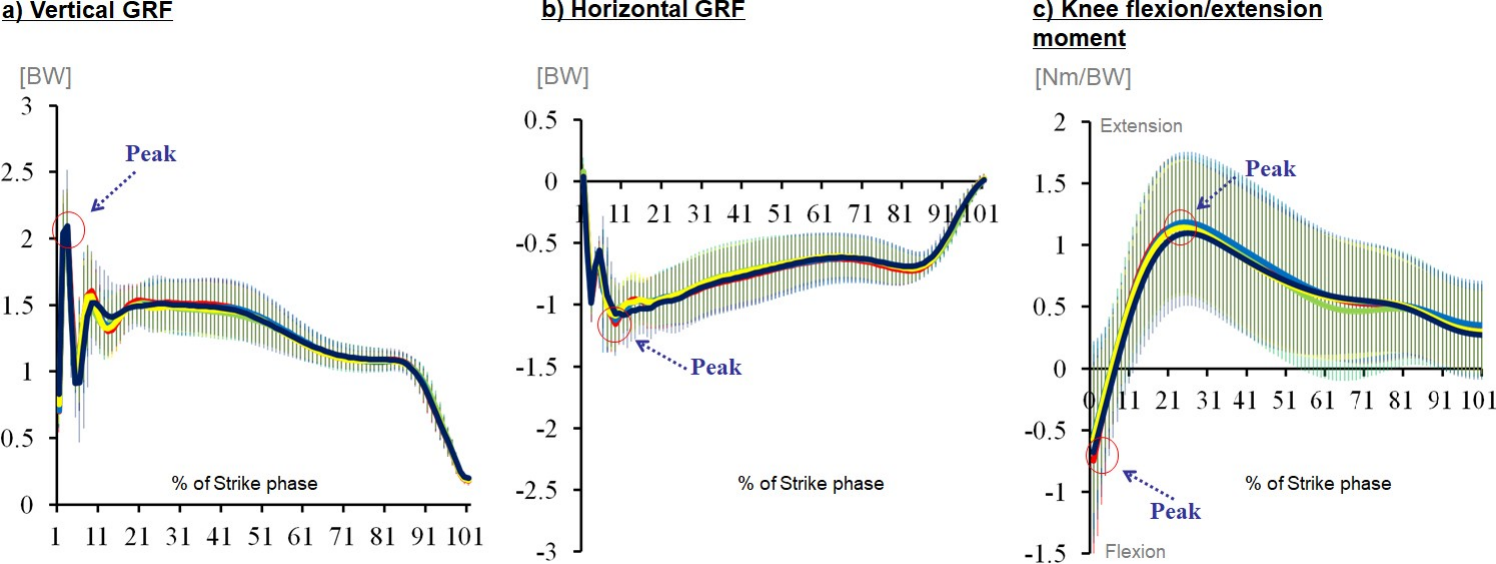
Typical curves of a) vertical GRF, b) horizontal GRF and c) knee flexion/extension moment.

**Fig 3:**
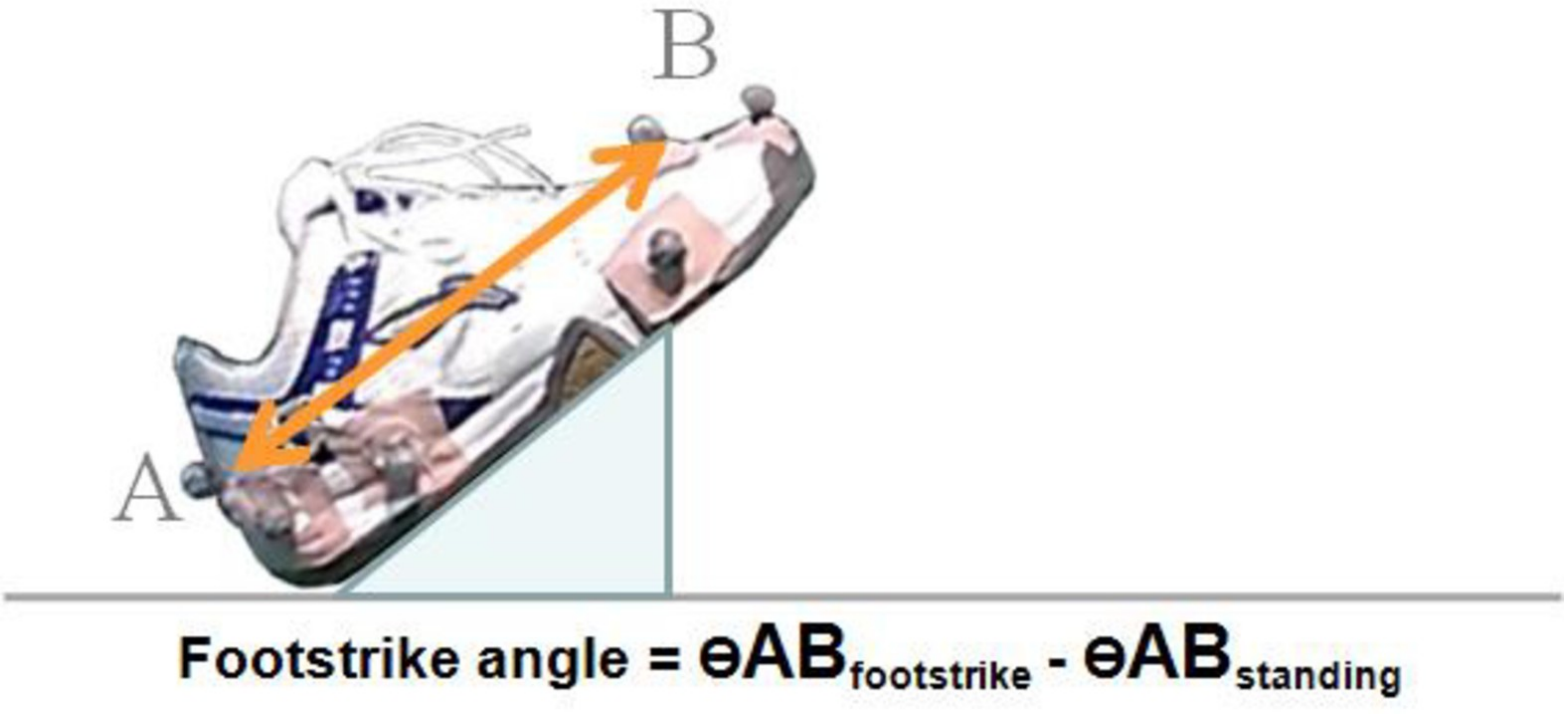
Definition of footstrike angle.

All marker trajectories and GRF signals were recorded at 200 Hz and 1,000 Hz, respectively. A spline interpolation was performed to rectify minor missing data using three frames of data before and after the missing data. The marker trajectories and GRF data were filtered with a fourth-order Butterworth bidirectional filter at 12 Hz and 100 Hz, respectively [13, 28]. The cut-off frequencies were determined by the smallest difference between the estimated and true signal values [30]. The contact phase of the lunge step was identified as the period from initial heel contact of the landing foot to toe-off, as determined by the force plate. The instance of heel contact and toe-off were defined as when the vertical GRF first exceeded 10N (heel contact) and reduced to 10N (toe-off). Peak vertical GRF, peak horizontal GRF, vertical maximum loading rate and knee moments were chosen for statistical analyses based on the previous literature linked to impact injuries in badminton lunge [13, 18, 31, 32]. In brief, peak vertical and horizontal GRFs are defined as the local maximum of the vertical and horizontal GRF, respectively (Fig 2). Vertical maximum loading rate is the maximum slope of the vertical GRF curve between the successive data points from 20% to 90% before the first peak impact [28, 33]. Mean loading rate was calculated from 0% to 100% of the peak vertical GRF [28, 33]. To allow comparison between groups, all variables related to GRF and knee moment data were normalized by the individual subject’s body weight (BW) and individual maximum lunge distance was normalized by the individual body height prior to further data processing [18]. Only knee sagittal plane moments were evaluated in the present study because the knee joint motion during lunge falls primarily in the sagittal plane, which contributes to the major knee biomechanical characteristics in badminton research [13, 18]. The local coordinate systems for the knee joint was defined by the proximal end of the tibia with an orientation based on the right hand coordinate system with the y-axis and z-axis directed vertically and posteriorly, respectively. External knee moment was calculated with inverse dynamics [18]. A positive value for joint moment denoted knee extension, with zero degree defined at neutral static standing position. In addition, approaching speed, foot contact time, and initial footstrike angle were measured to describe the general movement characteristics across groups of participants (Fig 3, [13, 28]). The approaching speed was defined as the averaged speed from the starting position to the initial contact of the force plate. The foot contact time was defined as the period of time from initial contact to final take-off of the lunging leg determined by the fore plate.

All statistical analyses were performed using SPSS 21.0 (IBM Corp., Armonk, NY, USA). Being robust to moderate violations of normality observed in most of the data, a 2 (gender) x 2 (skill-level) factorial ANOVA was performed to investigate if there were any significant interaction, gender and level effects for peak vertical GRF, peak horizontal GRF, loading rate, knee moment and movement characteristics variables. Level of significance was set at 0.05. Greenhouse-Geisser’s epsilon adjustment was used in all cases when Mauchly’s test indicated that the sphericity assumption had been violated.

## Results

### Movement kinematics

Two-way factorial ANOVA revealed significant main effects of gender and skill-level (*P* < 0.01), but not the interaction between gender and skill-level on approaching speed, foot contact time, footstrike angle and lunge distance (*P* > 0.05) (Table 2). Male athletes had faster approaching speed (*P* < 0.001; male 3.87 and female 1.08 m/s) and longer maximum lunge distance (*P* < 0.001; male 1.47 and female 1.16 body height) compared with female athletes. Unskilled athletes had smaller footstrike angle (*P* < 0.001; skilled 45.8 and unskilled 32.4°) but longer foot contact time (*P* = 0.02; skilled 0.69 and unskilled 0.75 s) compared with skilled athletes.

**Table 2:** Movement characteristics during each subject group in mean (standard deviation). BH = body height, *η*^*2*^ = partial eta squared; *β* = observed power. Significant P-values (< 0.05) are shown in bold. (raw data found in S1 File).

		Level		Interaction			Gender			Level		
	Gender	Skilled	Unskilled	*P*	*η*^*2*^	*Β*	*P*	*η*^*2*^	*β*	*P*	*η*^*2*^	*β*
Approaching speed (m/s)	Male	3.37 (1.22)	4.37 (2.24)	.21	.03	.24	**< .001**	.54	1.00	.17	.04	.28
	Female	1.05 (0.14)	1.10 (0.11)									
Foot contact time (s)	Male	0.71 (0.14)	0.78 (0.10)	.83	< .01	.06	.07	.07	.45	**.02**	.11	.68
	Female	0.66 (0.07)	0.72 (0.08)									
Footstrike angle (°)	Male	44.59 (5.05)	31.93 (5.84)	.66	< .01	.07	.36	.02	.15	**< .001**	.55	1.00
	Female	46.97 (7.19)	32.77 (6.75)									
Maximum lunge distance (BH)	Male	1.49 (0.10)	1.44 (0.09)	.17	.04	.28	**< .001**	.77	1.00	.67	< .01	0.07
	Female	1.14 (0.09)	1.17 (0.08)									

### Ground reaction force variables

**Fig 4:**
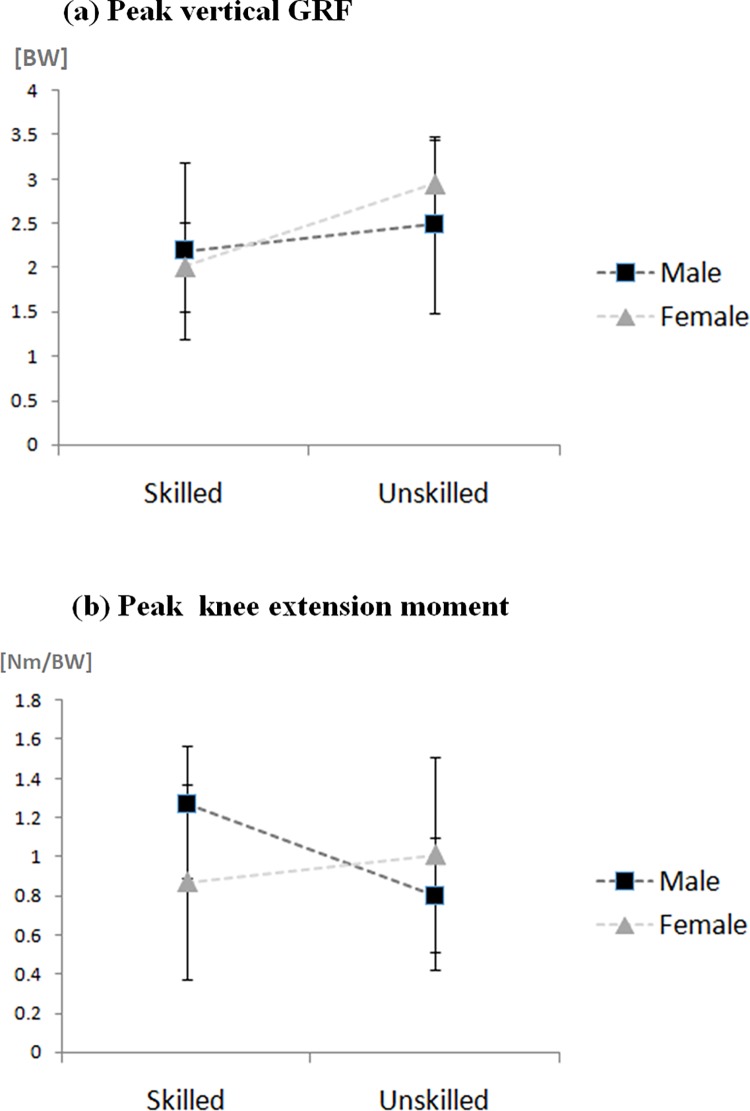
Interaction effect of a) peak impact and b) knee moment.

Interactions of gender x skill-level were found in peak vertical GRF (*F*(1,48) = 6.86, *P* < 0.05) (Table 3 & Fig 4A). The simple main effect indicated that female unskilled athletes experienced greater peak vertical GRF (*P* < 0.001; female skilled 2.01 and female unskilled 2.95 BW) than the female skilled athletes, while there was no significant difference between male skilled and unskilled athletes (*P* = 0.09; male skilled 2.19 and male unskilled 2.49 BW). In addition, the gender effect indicated that male athletes exhibited larger maximum loading rate (male 215.7 and female 121.65 BW/s) and mean loading rate (male 178.43 and female 81.77 BW/s) compared to female athletes (*P* < 0.001). The skill-level effect also indicated that unskilled athletes exhibited greater peak vertical GRF (*P* < 0.001; skilled 2.10 and unskilled 2.72 BW), greater peak horizontal force (*P* < 0.001; skilled 1.61 and unskilled 2.40 BW) but smaller mean loading rate (*P* < 0.01; skilled 150.15 and unskilled 110.05 BW/s) compared to skilled athletes.

**Table 3:** Ground reaction force characteristics during each subject group in mean (standard deviation). BW = body weight; *η*^*2*^ = partial eta squared; *β* = observed power. Significant *P*-values (< 0.05) are shown in bold. (raw data found in S1 File).

		Level		Interaction			Gender			Level		
	Gender	Skilled	Unskilled	*P*	*η*^*2*^	*β*	*P*	*η*^*2*^	*β*	*P*	*η*^*2*^	*β*
Peak vertical GRF (BW)	Male	2.19 (0.34)	2.49 (0.48)	.**01**	.13	.73	.26	.03	.20	**< .001**	.35	.99
	Female	2.01 (0.20)	2.95 (0.56)									
Peak horizontal force (BW)	Male	1.65 (0.34)	2.47 (0.45)	.76	< .01	.06	.33	.02	.16	**< .001**	.49	1.00
	Female	1.57 (0.33)	2.32 (0.48)									
Maximum loading rate (BW/s)	Male	229.23 (55.93)	202.17 (15.15)	.05	.08	.50	**< .001**	.57	1.00	.79	< .01	.06
	Female	112.59 (13.32)	130.70 (27.33)									
Mean loading rate (BW/s)	Male	195.27 (43.26)	161.59 (70.20)	.61	.01	.08	**< .001**	.55	1.00	**< .01**	.17	.88
	Female	105.03 (18.21)	58.51 (20.68)									

### Knee kinetic variables

The ANOVA indicated the significant interaction between gender and level on peak knee extension moment (Table 4 & Fig 4B, *F*(1,48) = 7.75, *P* < 0.01). The simple main effect indicated that male skilled athletes experienced greater peak knee extension moment than the male unskilled athletes (*P* < 0.001; male skilled 1.27 and male unskilled 0.80 Nm/BW), while there was no significant difference between female skilled and unskilled athletes (*P* > 0.05; female skilled 0.87 and female unskilled 1.01). For peak flexion moment, the male athletes elicited larger peak knee flexion moment compared with female (*P* < 0.001; male 0.75 and female 0.69 Nm/BW) and the unskilled athletes demonstrated larger peak knee flexion moment than the skilled athletes (*P* < 0.05; skilled 0.69 and unskilled 0.75 Nm/BW).

**Table 4:** Knee moments during each subject group in mean (standard deviation). BW = body weight; *η*^*2*^ = partial eta squared; *β* = observed power. Significant *P*-values (< 0.05) are shown in bold. (raw data found in S1 File).

		Level		Interaction			Gender			Level		
	Gender	Skilled	Unskilled	*P*	*η*^*2*^	*β*	*P*	*η*^*2*^	*β*	*P*	*η*^*2*^	*β*
Peak knee extension moment (Nm/BW)	Male	1.27 (0.55)	0.80 (0.30)	**< .01**	.14	.78	.40	.02	.13	.14	.05	.32
	Female	0.87 (0.32)	1.01 (0.38)									
Peak knee flexion moment (Nm/BW)	Male	0.71 (0.14)	0.78 (0.10)	.46	.01	.11	**< .001**	.49	1.00	**.03**	.10	.60
	Female	0.66 (0.07)	0.72 (0.08)									

## Discussion

While excellence of a lunge is the key success in badminton [25], peak vertical and horizontal forces experienced from it may be up to three times and two times of an individual’s body weight, respectively [13, 14]. This would place a considerable amount of stress on the lower extremities of athletes [14]. The present study compared the impact and knee joint loading across badminton athletes of different gender and skill-levels. The main findings in the present study were: (1) Male athletes had faster approaching speed, longer maximum lunge distance, larger maximum and mean loading rate and larger peak knee flexion moment compared with female athletes; (2) Unskilled athletes exhibited smaller footstrike angle, longer contact time, larger peak horizontal GRF, smaller mean loading rate and larger peak knee flexion moment than the skilled athletes; (3) the interaction indicated greater peak GRF impact in female unskilled athletes compared with female skilled athletes, while there was no difference between male participants. The results support the hypothesis that less-skilled and/or female athletes would experience a higher GRF and knee loading during an extreme lunge.

### Gender effect

It has been shown that female athletes had higher knee injury rates than male athletes in many sports such as basketball and soccer [34]. The present results demonstrated faster approaching speed and longer maximum lunge distance for male compared to female athletes, which is related to stronger musculature and strength in male athletes [9]. The higher loading rates in male athletes could be influenced by many factors including speed, initial footstrike pattern and contact time [23, 35]. Additionally, the present results indicated that male athletes exhibited larger loading rates and peak knee flexion moment compared to female athletes. The gender differences of kinetics results in present study, however, opposed the gender differences in the studies on cutting [20] and landing manoeuvres [36] which suggested that female athletes are susceptible to a higher risk of ACL injury as indicated by higher knee loading. One plausible explanation is the difference in the movement plane of study, which was executed predominantly in the anterior-posterior direction of a lunge step, compared with cutting and landing that was executed in the medial-lateral and vertical directions, respectively [16]. The other explanation would be the differences in test variables (i.e., GRF loading versus knee kinetics) and/or approaching speed differences between genders. Considering that increased knee valgus angle and moment during landing are thought to be the risk factors of ACL injury in badminton [16, 37], further investigation of neuromuscular control should be carried out to ascertain if male athletes’ movement would lead to greater impact loading on the lower extremities.

### Skill effect

The current findings indicated that skilled athletes had larger footstrike angles but shorter foot contact times compared to unskilled athletes, which is partially supported with a previous study [19]. Footstrike angles together with postural control, leg stiffness and muscle activation may influence the effect of cushioning on impact forces [38]. The human could produce the optimal movement control to avoid impact forces during lunge.

The current results also indicated that unskilled athletes exhibited greater peak horizontal GRFs and greater peak knee flexion moments, but smaller mean loading rates compared to skilled athletes. Higher knee flexion/extension moments were associated with greater quadriceps forces and tibial shear forces [16, 39], implying that unskilled athletes may be exposed to higher risk of overuse injuries. One possible explanation would be due to the physical and motor control characteristics, such as skilled badminton athletes being better quantity and quality of muscle properties and recruitment for stronger muscular strength compared to unskilled athletes’ characteristics [9]. Body somatotype characteristics increase proportionally through training level and intensity [40]. Another possible explanation would be that high skilled athletes would optimise the stretch-shorten cycle to increase the efficiency of a sequential proximal-distal joint action chain (i.e. smaller knee flexion moment) for badminton movements [40, 41]. Furthermore, the better efficiency of movement executed by skilled players is expected to reduce fatigue and impact levels to the body [9, 15, 42]. Thus, studying physiological profiles, muscle activation and neuromuscular control should be investigated further to identify the differences among playing levels [4, 12, 21, 43].

### Interaction between gender and level

The simple main effects analysis of the interaction indicated that female unskilled athletes experienced greater peak vertical GRF than female skilled athletes, while no difference of impact loading was found between male skilled and unskilled athletes. It would be possible that the larger skilled-unskilled difference (e.g. muscle strength, movement control efficiency) was found in females compared with males. Female unskilled athletes might have inferior movement efficiency, which may result in poorer impact attenuation by the body [9, 15, 42]. Repetitive high impacts would be associated with common badminton injuries such as patellar tendionosis, anterior cruciate ligament (ACL) and Achilles tendon injuries [10, 14, 17] that can lead to decreased participation time and level of performance as well as massive medical expenses [44, 45]. In the future, more attention should be paid when designing training and rehabilitation regimes for female athletes of various playing levels.

### Implications

Lunge movements are essential and frequently executed in badminton games. Since there is not much information on how impact loading may be related to injuries, the ground reaction force and joint loading profiles presented in the present study can serve as a reference for coaching and injury prevention. For example, due to stronger musculature and strength in male athletes [9], male athletes showed faster approaching speed and longer maximum lunge distance compared to female athletes. This could be related to higher loading rates of peak vertical GRF and peak knee flexion moment. Future footwear and orthosis development should consider greater emphasis on male data. More research is warranted to understand how structural changes in footwear may influence performance and the risks of injury in badminton players.

Greater peak horizontal GRF and peak knee flexion moment among unskilled athletes was another important finding. This implicates that unskilled athletes may be exposed to higher risk of overuse injuries [39] and that increase movement efficiency of a sequential proximal-distal joint action chain through training could be associated with smaller joint loading [40, 41]. Considering that the ability to attenuate repetitive high impacts is important to reduce the risks of overuse [10, 14, 17], more attention should be paid when designing training and rehabilitation regimes for athletes of various playing levels in the future.

### Limitation

When interpreting our results, it is important to consider several limitations in our study. First, individual maximal-effort lunges tested may have caused differences in initial boundary condition that might exhibit distinct landing kinematics and loading responses. The kinetic responses should be revisited under the same control speed condition. Second, only the lunge movement was measured and hence our findings may not be generalised to other badminton movements such as smashing and high clear which also involve considerable vertical landing loads. Third, the lunge movements were investigated in a well-controlled laboratory environment. The realistic situation (i.e., movement intensity, motivation, reactive response to the opponent) should be incorporated in future studies. In a recent study on badminton lunge, isolated and repeated lunge trials displayed distinct mechanisms of loading response and adaptation during impact attenuation phase of a lunge step [13]. Future study is warranted to investigate the impact loading among groups at various movement intensities and motivations.

## Conclusions

Badminton athletes of various gender and skill-level exhibited distinct landing kinematics during a badminton lunge. Male athletes experience greater impact loading rate and knee flexion moment while unskilled athletes experienced both greater vertical and horizontal GRF loading, and knee flexion moment during badminton lunge. In addition, unskilled female athletes seem to be more vulnerable to lower extremity injuries as they experience higher impacts during landing compared to the other three groups of players. This information would help in the designing of training and rehabilitation regimes for better protection against impact-related injuries in badminton.

## Supporting information

S1 File Provides supporting information for Tables 1 to 4, respectively. (XLSX)
